# Identifying and adapting interventions to reduce documentation burden and improve nurses’ efficiency in using electronic health record systems (The IDEA Study): protocol for a mixed methods study

**DOI:** 10.1186/s12912-022-00989-w

**Published:** 2022-08-04

**Authors:** Gillian Strudwick, Lianne Jeffs, Jessica Kemp, Lydia Sequeira, Brian Lo, Nelson Shen, Petroiya Paterson, Noelle Coombe, Lily Yang, Kara Ronald, Wei Wang, Sonia Pagliaroli, Tania Tajirian, Sara Ling, Damian Jankowicz

**Affiliations:** 1 Institute of Health Policy, Management and Evaluation, University of Toronto, Toronto, ON Canada; 2 Centre for Complex Interventions, Centre for Addiction and Mental Health, 60 White Squirrel Way, Toronto, ON M6J 1H4 Canada; 3 Information Management Group, Centre for Addiction and Mental Health, Toronto, ON Canada; 4 Lunenfeld-Tanenbaum Research Institute, Sinai Health, Toronto, ON Canada; 5 Bloomberg Faculty of Nursing, University of Toronto, Toronto, ON Canada; 6 Canada Health Infoway, Toronto, ON Canada; 7 Clinical Informatics, Sinai Health, Toronto, ON Canada; 8 Quality and Patient Experience, Sinai Health, Toronto, ON Canada; 9 Professional Practice, Nursing and Health Disciplines, Sinai Health, Toronto, ON Canada; 10 Cerner Canada, Markham, ON Canada; 11 Department of Family and Community Medicine, Faculty of Medicine, University of Toronto, Toronto, ON Canada

**Keywords:** Nursing, Electronic health records, Documentation, Burnout, Efficiency, Nursing informatics, Clinical informatics, Health information technology

## Abstract

**Background:**

Although EHR systems have become a critical part of clinical care, nurses are experiencing a growing burden due to documentation requirements, taking time away from other important clinical activities. There is a need to address the inefficiencies and challenges that nurses face when documenting in and using EHRs. The objective of this study is to engage nurses in generating ideas on how organizations can support and optimize nurses’ experiences with their EHR systems, thereby improving efficiency and reducing EHR-related burden. This work will ensure the identified solutions are grounded in nurses’ perspectives and experiences and will address their specific EHR-related needs.

**Methods:**

This mixed methods study will consist of three phases. Phase 1 will evaluate the accuracy of the EHR system’s analytics platform in capturing how nurses utilize the system in real-time for tasks such as documentation, chart review, and medication reconciliation. Phase 2 consists of a retrospective analysis of the nursing-specific analytics platform and focus groups with nurses to understand and contextualize their usage patterns. These focus groups will also be used to identify areas for improvement in the utilization of the EHR. Phase 3 will include focus groups with nurses to generate and adapt potential interventions to address the areas for improvement and assess the perceived relevance, feasibility, and impact of the potential interventions.

**Discussion:**

This work will generate insights on addressing nurses’ EHR-related burden and burnout. By understanding and contextualizing inefficiencies and current practices, opportunities to improve EHR systems for nursing professional practice will be identified. The study findings will inform the co-design and implementation of interventions that will support adoption and impact. Future work will include the evaluation of the developed interventions, and research on scaling and disseminating the interventions for use in different organizations, EHR systems, and jurisdictions in Canada.

## Background

Nurses represent the largest group of healthcare providers in Canada and have been reported to be the primary users of EHR systems [1, 2]. While EHR systems serve as the backbone of documentation for all clinical activities [3], studies have shown that nurses spend a greater amount of time documenting their assessments, care, and outcomes in the EHR now than in the past [4, 5]. While previous initiatives have focused on expanding the design and functionalities of EHRs for research purposes to support clinical decision-making and assist with patient risk assessments [6–9], the growing burden caused by documentation requirements has overshadowed these efforts and led to inefficiencies in the EHR, taking time away from patient care activities and reducing joy in the profession [7, 9–14].

EHR systems are essential and provide value to nurses in several ways. For example, clinical decision support systems and barcode medication administration have been shown to support patient safety and reduce potential harm in nursing care [15]. However, these gains are greatly diminished by the significant investment into the system by individual nurses (e.g., time spent in the EHR, number of clicks) [11, 14]. Existing literature has included studies identifying the challenges nurses face when utilizing EHRs in their practice for documentation [9, 14–20]. These challenges include but are not limited to, poor design and usability [20], working with hybrid (paper and electronic) systems requiring complex workflows [21], duplicate data entry [22, 23], multiple system logins, difficulty in finding the ‘patient story’ or specific patient information [24], propagation of errors throughout the record, too many alerts [25], and poor navigation [10]. These factors contribute to high documentation burden and low system usability, which commonly results in poor satisfaction with the EHR among nurses [22, 26, 27].

Recent studies and reports point to increasing documentation demands and data entry among nurses, especially as nursing data is used for purposes outside of care provision (e.g., reporting and research) [6, 7, 24]. Other studies involving providers of various professional backgrounds have described that providers feel the time spent using EHRs takes away from patient care [28, 29], and they do not always find that the system provides greater efficiency and a reduction in administrative tasks [30, 31]. EHRs can also add to the cognitive load of providers due to high documentation requirements [28, 29, 32, 33]. This burden on time and resources associated with using the EHR has been recognized as one of the many contributors to burnout among providers [20, 34]. Through surveying physicians at one of the sites within the proposed study, 74.5% of respondents who were burned out (~ 25.6%) identified the EHR as a contributor to their burnout [20]. Additionally, prior to the pandemic, it was found that burnout among nurses was higher than physicians (34%) [20]. This finding aligns with numerous opinions, viewpoints and other literature discussing this topic [30, 35–39] — all identifying the need to understand how the burden of EHR use may contribute to the multifactorial issue of burnout. If burnout is not addressed, it can lead to career dissatisfaction [36], absenteeism and job turnover [37], reduced quality of care [38], and medical errors [39].

### Study objective

The objective of this study is to engage nurses to understand their experiences using EHR systems and generate ideas on how to optimize their experiences with the goal of improving efficiency and reducing EHR-related burdens. In this context, efficiency is defined as the summative number of clicks (‘click burden’) in the system and the time required to complete certain tasks in the EHR. This work will ensure that the identified solutions are grounded in nurses’ perspectives and experiences and will address their EHR-related needs.

## Methods

This study will generate ideas and develop meaningful interventions to support and optimize nurses’ use and experiences with the EHR system through the following aims:

1. Evaluate the utility of the analytics platform to accurately capture the EHR utilization patterns of nurses.
2. Understand the utilization patterns and user experiences of nurses in the EHR.
3. Identify areas for improvement in the utilization of the EHR for documentation.
4. Generate and adapt potential interventions to improve the efficiency of nurses’ EHR use.
5. Rank and reduce intervention options through nurses’ assessment of the relevance, feasibility, and perceived impact.

### Participants and settings

This study will be conducted at two hospital sites in Toronto, Canada. One site is Canada's largest mental health and addictions teaching hospital and academic centre. This organization offers care to individuals with mental health and addiction needs through virtual, inpatient and outpatient care, as well as a partial hospital/day treatment area, and a psychiatric emergency department. There are approximately 900 actively practicing nurses at the organization. Nursing staff work in all clinical areas of the organization and complete all documentation within the EHR system. Inpatient and outpatient clinical areas, excluding the emergency department, will be the setting in which the study will take place at this site.

The second site is an acute care hospital and academic health science centre with clinical areas including women’s and infants’ health, emergency medicine, cancer, complex orthopaedics, palliative care, diabetes, inflammatory bowel disease, geriatrics, and arthritis and autoimmune disease. This site employs more than 1200 nurses working in all areas of the organization and nurses complete the majority of their documentation within the EHR. The women’s and infants’ inpatient department will be the setting in which the study will take place at the second site. A list of study sites can be obtained from the corresponding author upon request.

Eligible participants in this study are 1) nurses (Registered Nurse, Registered Practical Nurse, Registered Psychiatric Nurse, or Nurse Practitioner); 2) employed in one of the two study settings; 3) providing direct patient care, and 4) documenting most of their notes in the EHR. Nurses at the first hospital site will be recruited through email communications sent to all nursing staff. The second hospital site will also conduct recruitment through email communications, but recruitment will be completed with a single inpatient program at the hospital (e.g., Women’s and Infants’ Health). If nurses are interested in taking part in the study, they will be able to contact the study team to join or obtain further information.

A Nursing Advisory Council (NAC) will be established and will include nurses from the relevant clinical areas at both hospital sites in which the study data will originate, who have differing levels of comfort using the EHR, and varied years of experience in nursing. The NAC will meet on a quarterly basis to provide guidance to the study team on the feasibility and applicability of research activities such as recruitment methods and data analysis. Both sites will use existing hospital structures to determine if nurses are interested in joining the NAC. Additionally, the study will be conducted collaboratively with the information technology/clinical informatics departments at each hospital to ensure that the proposed interventions are feasible and can directly inform current EHR optimization initiatives.

### Study design

**Fig. 1:**
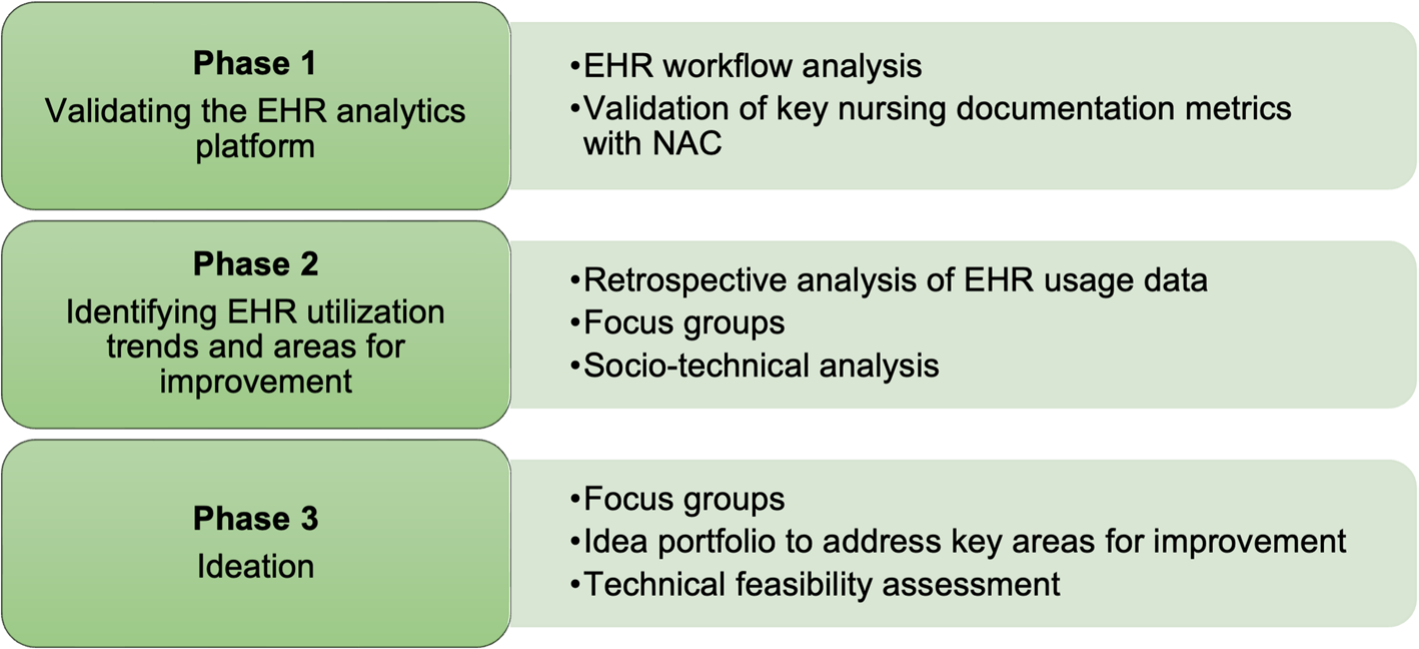
Overview of methods used in each phase of the mixed methods study

The research aims will be addressed through three phases of the study (Fig. 1) utilizing a mixed method design following Palinkas et al.’s taxonomy of designs in implementation research [40]. The taxonomy has three elements to study design including structure, function, and process [40]. For the purpose of this study, the Quan to QUAL structure will be used; this is defined by a sequential process of data collection and analysis that begins with quantitative data and is followed by qualitative data collection. The main purpose of the quantitative data collection and analysis is to test the hypothesis that nurses are spending an abundance of their time in the EHR, mainly on documentation. Based on Palinkas et al.’s taxonomy, the function of the study design will follow complementarity methods; this type of function uses both quantitative and qualitative data to address the set of aims of the study [40]. In this case, quantitative data will be used to evaluate various outcomes of the analytics platform and identify utilization patterns, and qualitative data will be used to provide a deeper understanding and contextualization of these results, as well as aid in the development of interventions. The study process will use the method of connection; meaning the quantitative data will be used to guide the qualitative focus group discussions [40].

#### Phase 1 – Validating the EHR analytics platform

The EHR system’s analytics platform will be tested and validated in a test EHR environment. The validation will be conducted to ensure that the system’s analytics are accurate in capturing nursing utilization metrics for multiple tasks (e.g., documentation, chart review and medication reconciliation). Key nurse EHR documentation metrics will be identified through a retrospective analysis of the nursing EHR usage data.

#### Phase 2 – Identifying EHR utilization trends and areas for improvement

Identification of trends in EHR utilization and inefficiencies based on key nursing EHR documentation metrics. EHR utilization trends will be identified using the quantitative data extracted from the nursing-specific EHR analytics platform. In order to examine the differences in EHR usage between part- and full-time nurses, a subgroup analyses (by role) will be conducted on the metrics. These trends will be shared with the NAC to validate results and determine key nursing documentation metrics. This phase will also include focus groups with nurses used to contextualize the trends and usage and identify processes for improvement. These focus groups will ensure that the proposed interventions are appropriate and developed with nursing staff.

#### Phase 3 – Ideation

The study team will conduct focus groups with nurses to co-create and rank potential solutions to address key areas for improvement based on processes identified in Phase 2. Nurses will also assess the relevancy, feasibility, and impact of potential interventions in addressing the identified gaps.

### Frameworks

This study will draw upon the Consolidated Framework for Implementation Research (CFIR), which outlines considerations for successful implementation projects [41]. The CFIR has been used successfully in the health informatics field to deliver actionable insights to drive optimization and implementation success. Specifically, the findings of Phase 2 will be mapped to the constructs of CFIR as shown in Table 1.

**Table 1:** Mapping of Study Components to the CFIR

*CFIR Domain*	*Use in Study*
Intervention Characteristics	*Adaptability*: Optimization of the organizations’ EHR systems is possible through working with in-house clinical applications/informatics teams and external vendor teams
Outer Setting	*External policies and incentives: *Organizational policies that are currently in place and govern nursing documentation and practice
Inner Setting	*Structural characteristics*: Health Information and Management Systems Society (HIMSS) designations (if applicable) *Organizational and unit culture*: the role of nurse managers in EHR adoption
Characteristics of Individuals	*Nurses’ experience with the EHR:* These experiences are dependent on the clinic and context nurses practice in (e.g., outpatient vs. inpatient), documentation requirements, and training *Individual identification with the organization*: Full-time vs. casual status, length of employment, and other EHR vendors nurses may have worked with in the past
Process	*Planning*: Phases 1 and 2 will correspond with the planning (i.e., mapping nurses’ experience to the EHR analytics platform) *Executing*: Phase 3 will correspond with executing through the development of initiatives

In addition to the CFIR, Sittig and Singh’s 8-dimensional Socio-technical Model will also be used for analysis during the study in Phase 2 (Table 2) [42, 43]. The Socio-technical Model will be used to build upon the five domains of the CFIR by considering the nuances of studying health information technologies and the complexities of healthcare systems [42].

**Table 2:** Sittig and Singh’s 8-Dimensional Socio-technical Model

*Dimension*	*Definition*
Hardware and software computing infrastructure	Technical dimension consisting of physical devices and digital applications to keep devices running
Clinical content	Structured or unstructured textual or numeric data or images stored in the system
Human–computer interface	Allows users to interact with the system by seeing, touching, or hearing
People	Humans involved in all aspects of the design, development, implementation, and use of health information technology
Workflow and communication	How people work together cohesively to accomplish patient care
Internal organizational policies, procedures, and culture	Organizations’ internal structures
External rules, regulations, and pressures	External forces that facilitate or place constraints on the design, development, implementation, use, or evolution of health information technology
System measurement and monitoring	Routine evaluation of the use, effectiveness, and outcomes of health information technology

### Data collection

Data used in Phases 1 and 2 of the study will be extracted from the nursing-specific EHR system’s analytics platform. This platform has previously been used to capture EHR utilization patterns for physicians at one of the study sites [20]. Prior to the initiation of Phase 2 focus groups, the NAC will be consulted to identify the subset of metrics to be validated from the complete list captured within the EHR analytics platform. The study team will document the raw time taken for each task within validation tests done at each site.

In Phase 2, a 12-month retrospective analysis of the nursing-specific EHR system’s analytics platform will be conducted to identify trends in utilization of the subset of metrics identified by the NAC. A list of all actively employed nurses (across the two study settings) for the duration of the 12-month period will be obtained. Since there is an aim to build interventions that improve the experience for nurses, all nurses will be included in the analysis who have used the EHR system during that period. As the metrics will be analyzed in aggregate (not at the individual level), nurses who are on leave (> 6 weeks) during the selected time period will not be contributing data to the analytics platform and will therefore not be included in the calculation for that period. Data will be collected on a monthly basis, consistent with one study site’s past analysis of physician data as well as studies by others that have completed similar analyses at comparable or larger scale (e.g., hospital network) [44, 45].

Following the analyses, an infographic summary of the results will be developed and used as a foundation for discussion in the first round of 60-min focus groups with nurses (Phase 2) [7, 46, 47]. The focus groups held during Phase 2 will be used to lead an open discussion about the trends in the analysis and to contextualize why the trends may be occurring. The second objective is for nurses to assess which processes associated with usage trends can be improved, the feasibility of improving the process, and the perceived impact it will make to their EHR experience. The focus groups will follow Kruger and Casey's methods [48]. A maximum variation sampling strategy will be used to recruit a variety of viewpoints and ensure that a diverse group of participants across sex, gender, age, and ethnicity are selected; however, each of the focus groups will consist of participants based on area of care (e.g., Women’s and Infants’ Health) and study site [49]. To achieve study objectives, approximately six focus groups with eight participants each will be conducted at each site. The estimated number of participants was determined using Palinkas et al.’s guidelines for meaningful sampling for qualitative data collection in mixed method implementation research [49]. To reach adequate participant enrolment, the study team will work with one of the sites equity and diversity offices to develop inclusive and effective recruitment materials as well as focus group guides.

In Phase 3, the purpose is to co-create a list of possible solutions to address the areas of improvement identified in Phase 2. A prioritization activity will be used to identify the top solutions. Data will be collected through a 90-min focus group using an online whiteboard platform (e.g., Miro) to allow the participants to collaborate in generating ideas. The focus group session will utilize service design methods incorporating *brainstorming, affinity mapping*, and an *idea portfolio* [17]. Through these methods, participants will brainstorm potential solutions, group similar solutions, and assess the solutions on their feasibility and impact. The focus group session will close with a discussion about the top-rated solutions and considerations for implementation.

Further details about data management processes used in this study, including security and storage, are available upon request from the corresponding author.

### Data analysis

In Phase 1, raw and percent differences will be calculated to estimate the amount of discrepancy between the two sources of data. The tests run by the study team will be conducted multiple times for each workflow to ensure test–retest reliability of the findings. Based on previous studies, [12, 19, 50, 51], an estimated discrepancy of < 2 min, or 10%, is considered reasonable for validity. If a discrepancy higher than these thresholds is found, the study team will work with the EHR vendor to identify potential causes.

In Phase 2, analyses will be completed and individual users that fall significantly outside of typical ranges will be assessed to determine if they should be removed from the dataset (e.g., their role differs significantly from typical nursing practice). The metrics used for analysis will be determined by the NAC. Similar to previous work with physicians [20], the analysis will consist of descriptive statistics depicted over months to show trends and will include stratification by various user characteristics. The study team will explore the potential to perform a latent class analysis to cluster users into groups, which would be useful when identifying what set of interventions may be beneficial to sub-nursing population groups.

The focus groups will be audio recorded and transcribed verbatim and the results will be analyzed using qualitative content analysis [52–54]—an approach commonly used by the study team. Specifically, directed content analysis will be used to deductively code the focus group data based on CFIR constructs and the inter-connected dimensions in Sittig and Singh’s seminal Socio-technical Model (Table 2) [42]. Inductive coding will be used for data that does not fit into the pre-existing list of factors, offering the opportunity to extend the model. Both inductive and deductive coding will be used within each domain to provide more specificity. Data will also be stratified by nursing practice and demographic variables to gain insights into the nuances and differences across characteristics.

In Phase 3, the *idea portfolio* created by focus group participants will be used to develop a final ranked list of proposed solutions based on the relevance and feasibility of each solution for use in future rounds of ideation and prototyping at each study site. Implementation recommendations for each proposed solution will also be mapped to the relevant dimensions of the CFIR to ensure considerations are made based on the characteristics of the solution, setting, and individuals involved in or impacted by the solution.

## Discussion

This study will support the understanding of usage patterns and documentation requirements of various nursing populations at two hospital sites and provide insights into how nurses’ EHR-related burden and burnout can be addressed. Through the provision of support for EHR-related burdens and the implementation of meaningful changes to the EHR system, the study team hopes to contribute to the greater utility of their organizations’ EHR systems for nursing professional practice. Improvements will be based on interventions co-created with nursing staff to ensure that solutions contribute to efficiency and respond to the existing needs of nurses. Additionally, given the multi-site study settings including an array of nursing disciplines and experiences with the EHR, the findings will have the potential to inform changes across different clinical areas, hospitals, jurisdictions, and EHR systems.

### Dissemination

Participants will be asked if they would like to receive any publications or outputs resulting from this work. This will be asked during the focus groups and a password -protected file containing email addresses will be kept for this purpose. Local nursing informatics groups and associations will also disseminate knowledge findings through national communication products such as webinars, newsletters, and open-access links as appropriate. A briefing note will be distributed to organizations to share knowledge and guide EHR improvements for broader nursing populations. This will include organizations such as nursing and informatics associations, local academic health sciences networks, nursing leadership networks, digital health agencies, EHR vendors, and local networks of Chief Information Officers, Chief Medical Information Officers, and Chief Nursing Information Officers.

## Data Availability

The datasets generated and/or analysed during the proposed study will be available from the corresponding author upon reasonable request.

## Abbreviations

EHR: Electronic health record
CFIR: Consolidated Framework for Implementation Research
HIMSS: Health Information and Management Systems Society
NAC: Nursing Advisory Council
CIHR: Canadian Institutes of Health Research
